# PW03-001 - PFAPA syndrome in Turkish children

**DOI:** 10.1186/1546-0096-11-S1-A227

**Published:** 2013-11-08

**Authors:** ED Batu, F Kara Eroglu, Y Bilginer, S Ozen

**Affiliations:** 1 Pediatrics, Hacettepe University Medical Faculty, Ankara, Turkey; 2 Pediatric Rheumatology, Hacettepe University Medical Faculty, Ankara, Turkey

## Introduction

Periodic fever, aphthous stomatitis, pharyngitis, and adenopathy (PFAPA) syndrome is a clinical entity of unknown etiology, characterized by periodic attacks of fever, associated with aphthous stomatitis, pharyngitis and cervical adenopathy.

## Objectives

The aim of this study was to evaluate the demographic, clinical, laboratory and genetic characteristics of PFAPA patients, and to compare the parameters between MEFV carriers and noncarriers.

## Methods

Seventy-one children were enrolled in the study diagnosed as PFAPA according to the previously published criteria and followed-up at the Pediatric Rheumatology Clinic of Hacettepe Children’s Hospital. Mutation analysis included MEFV gene mutations. The ethical committee of our institute approved the study protocol.

## Results

The median age of onset was 12 months, with a male-to-female ratio of 1,6:1. The length of fever attacks ranged between 2-8 days, and symptom-free intervals between 10-45 days. The most common associated symptoms were pharyngitis (100%), abdominal pain (45,1%), aphthous stomatitis (43,7%), and cervical lymphadenitis (32,4%). During fever flares, the leukocyte count and acute phase reactants (ESR and CRP) were significantly higher (p<0,001). Of 53 patients tested for MEFV mutations, 2 were homozygote, while 20 had a single MEFV mutation (9 of them with M694V mutation). Thus carrier frequency reached 1:2,6, higher than the overall carrier frequency in healthy Turkish population which is 1:5 (p=0,037). Also, nearly half of these patients had a M694V mutation (associated with higher clinical severity in FMF) which is around 15% in healthy Turkish carriers. No differences were found between carriers and noncarriers in demographic data, clinical and laboratory parameters and response to therapies. In 37 of the 38 who received steroids, attacks were abrupted. The attacks recurred in only 1 out of 10 patients who underwent tonsillectomy. We have also shown a positive family history of recurrent pharyngitis in 52,1% of patients.

## Conclusion

The MEFV carrier frequency and M694V was significantly higher as compared to the healthy population. The high family history supports the heritability in PFAPA pathogenesis. PFAPA is a polygenic disease; the association with MEFV mutations in certain ethnic groups may suggest their possible role through the innate immune system. Studying the role of inflammasome in PFAPA may shed light on etiopathogenesis of PFAPA syndrome.

## Disclosure of interest

None declared

